# Controlling Posture and Vergence Eye Movements in Quiet Stance: Effects of Thin Plantar Inserts

**DOI:** 10.1371/journal.pone.0143693

**Published:** 2015-12-04

**Authors:** A. Foisy, C. Gaertner, E. Matheron, Z. Kapoula

**Affiliations:** IRIS team, Physiopathologie de la Vision et Motricité Binoculaire, FR3636 Neurosciences CNRS, Paris, France; State University of New York Downstate Medical Center, UNITED STATES

## Abstract

The purpose of this study was to assess properties of vergence and saccade eye movements as well as posture in quiet stance, and the effects of thin plantar inserts upon postural and oculomotor control. The performances of 36 young healthy subjects were recorded by a force platform and an eye tracker in three testing conditions: without plantar stimulation, with a 3 millimetre-thick plantar insert, either a Medial or a Lateral Arch Support (MAS / LAS). The results showed a decrease of the Surface and Variance of Speed and a more posterior position of the CoP with either stimulation compared with the control condition. The fractal analysis showed a decrease with MAS. Wavelet analysis in the time-frequency domain revealed an increase in the Cancelling Time of the low frequency band with MAS. These results suggest a better stability for a lower energy cost. Concerning eye movements, the inserts influenced only vergence (not saccades): MAS caused an increase of the phasic amplitude of divergence, and conversely a decrease of the tonic amplitude. In contrast, LAS caused an increase of the tonic amplitude of convergence. Thus, MAS renders divergence less visually driven, while LAS renders convergence more visually driven. We conclude that the CNS uses the podal signal for both postural and vergence control via specific mechanisms. Plantar inserts have an influence upon posture and vergence movements in a different way according to the part of the foot sole being stimulated. These results can be useful to clinicians interested in foot or eye.

## Introduction

Postural control is ensured by a multisensory system wherein eye and foot play an important role [1, 2]. Several studies, summed up in the systematic review from Christovao et al. [3], report the effects of different kinds of insoles on balance: vibrating insoles, textured insoles, quick-comfort insoles, insoles with spikes, flat insoles with different hardness, insoles with wedges and sensors and balance-enhancing insoles. Yet, none used thin (< 4mm) specific plantar inserts, which are used by some clinicians in an attempt to improve postural control of patients. The latter are appreciated for their many advantages: they are very thin and easy to wear contrary to insoles with wedges, they do not need any battery and they are customizable. Previous studies showed that thin plantar inserts can induce neuromuscular responses [4, 5], variations of plantar pressure distribution [6], of the medio-lateral position of the Center of Pressure (CoP) [7], or of foot eversion during walking [8]. Yet, the effects of such thin stimulations on balance in quiet stance have been little studied so far. Only an abstract of Janin and Toussaint [9] suggests that they can decrease sway area, and this experiment was done in a gaze fixation condition. No study using specific thin plantar inserts was ever realized while the eyes were moving and none used the new methods of analysis of the CoP displacements.

Besides, Roll and Roll [1, 2] showed that vibration of extraocular, but also neck or extrinsic foot muscles gives rise to an impression of displacement of a visual target while the subject is held stationary. The authors conclude that the proprioceptive cues arising from all the body segments are used to build a body reference, and that the absolute position of eye in space could be calculated thanks to this “proprioceptive chain” which links foot and eye. Yet, these experiments were done in monocular vision and the authors did not record eye movements. Hence, it was impossible to say if the impression of displacement of the visual target was linked to an actual movement of the eye or to a mere integrative phenomenon: the displacement of the visual target being the most logical interpretation of the perceived afferents. Later, Han and Lennerstrand [10], Lennerstrand et al. [11] and Popov et al. [12] underscored an actual version of the eye during neck muscle vibration. Erkelens et al. [13] compared the accuracy of vergence movements of standing subjects either with the target moved by the experimenter, or moved manually by the subject, or when the subject performed fore-aft movements of the torso. This last condition showed the greatest accuracy, suggesting that the paravertebral muscle’s proprioception helped in the performance. Han and Lennerstrand [14] also observed faster accommodative vergence movements during vibration of neck muscles. All those studies suggest that neck, torso and foot proprioception can act upon eye movements, in particular vergence. However, none of them got directly interested in the influence of plantar cutaneous afferents upon fine spatial and temporal parameters of vergence and saccades in standing subjects. Note that in everyday life we do make frequently those movements during visual exploration of 3D space while standing. It is likely that plantar exteroception can also modify eye movements. First, the two modalities of somesthesia are functionally linked and participate together in postural control [15–18]. Second, in the experiment of Erkelens et al. [13], the movements of the torso must have changed the repartition of the plantar pressures and therefore the plantar cutaneous afferents, which may have influenced the results. In addition, clinical observations and personal communications [19, 20] reported that thin plantar inserts entail modifications of phorias.

So far, the effects of the manipulation of podal or ocular inputs upon postural or oculomotor control have been studied separately, and vergence eye movements have been assessed almost exclusively in seated subjects. Yet, we permanently perform those movements while standing during various ecologic activities. Therefore our goal is to study vergence under such conditions during which the CNS must control at the same time ocular vergence and equilibrium, a complex but physiologic task. We wondered if and how the manipulation of podal afferents would influence both posture and eye movements.

**Fig 1:**
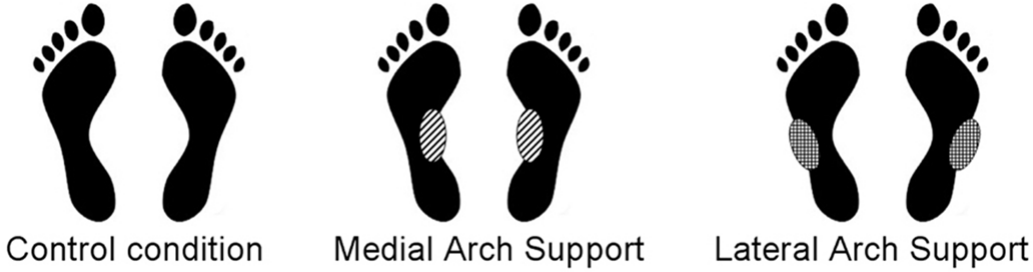
Posturography testing conditions. 3 mm thick inserts (SH 60A, 250 kg/mm²) were placed in the midfoot region: on the medial half of the external band of the footprint (MAS condition), or on the lateral half (LAS condition).

Our first hypothesis (hyp.1) concerns postural control: relying on the results of Janin and Toussaint [9] we expect that a bilateral Medial or Lateral Arch Support (see Fig 1) would improve postural control, especially the Lateral Arch Support.

Our second hypothesis (hyp.2) concerns oculomotor control: there is strong evidence that vergence improves postural control [21–24], while saccades entail small changes in postural control (only a mild decrease of the standard deviation of the antero-posterior sway–see [25]), hence, we hypothesize that the plantar stimulations will act upon vergence neural networks only. Moreover, saccade is a significantly faster movement than vergence (50 milliseconds v.s 300–400 –see [26]) and podal inputs need between 100 and 200ms to be treated [16, 27], which does not seem compatible with an influence upon saccades.

Furthermore, according to Roll and Roll [1, 2], some muscular groups have a synergic functioning: the invertors muscles and the ipsilateral convergence muscles seem to work together, and conversely for the evertors and divergence muscles. As the stimulation of the medial part of the foot sole favors inversion, and eversion for the lateral part [6, 28], we further hypothesize (hyp.3) that the Medial Arch Support would increase the amplitude of convergence, and the Lateral Arch Support would increase divergence.

Besides theoretical interest of the question, there is an important clinical context around the experiment. Many patients suffer from mechanical pain or diseases that are correlated to postural impairment [29–34] and it is common in clinical practice to use such inserts to take care of patients suffering from postural deficiency [35]. Better understanding of the action of these plantar inserts could contribute to improving their medical care.

## Materials and Methods

### Subjects

Thirty-six healthy young subjects, recruited in paramedical schools took part in the experimentation: 15 males and 21 females all in the age range 20–35 years (mean age = 25, 7 ± 3, 6 years, mean height = 170.81 ± 9.34 cm, mean body weight = 65.64 ± 10.73 kg).

None of them were taking medication and all of them were asymptomatic and had normal postural performance within the standard stabilometric values of the Association Française de Posturologie (AFP), that is the medio-lateral (X) mean position of the CoP included between -10 and +12 mm, Surface of CoP excursions between 39 and 210 mm², Variance of speed between 3 and 18 mm²/s².

All subjects were emmetropic and wore no glasses. Their visual acuity at close distance was examined by means of Parinaud’s reading test. The results were all normal (2 for 33 subjects, 3 for 3 of them). Binocular visual function was also assessed with the stereo acuity TNO test and all values were normal, that is 60” of arc or lower. We also measured the Near Convergence Point, which was 5.07 ± 2.19 cm, and the amplitude of accommodation with the push-up method (we did a mean of 3 measures for both tests) [36, 37]. The subjects had a mean of 9.15 dioptres (±1.61), which is within Duane’s normative data (9.5 ± 2 dioptres) [36]. The t-test test did not show any statistical difference with that theoretical physiologic value (p = .20).

### Ethics Statement

The investigation adhered to the principles of the Declaration of Helsinki and was approved by the ‘‘Comité de Protection des Personnes” (CPP) Ile de France VI (No: 07035), Necker Hospital, in Paris. The subjects gave informed written consent after the nature of the procedure was explained.

Their characteristics are summarized in Table 1.

**Table 1:** Subject’s characteristics.

Subject	Age	Gender	Height (cm)	Body weight (kg)	TNO	Parinaud (left/right)	Amplitude of Accom-modation (dioptres)	Near Convergence Point (cm)
S1	31	F	172	74	30	2/2	8,33	4,50
S2	25	M	173	71	30	2/2	12,05	1,40
S3	33	F	163	51	30	2/2	8,96	5,03
S4	22	F	168	62	60	2/2	8,88	6,13
S5	23	M	184	75	60	2/2	9,32	7,00
S6	22	F	172	66	30	2/2	10,20	8,03
S7	22	F	156	58	30	2/2	8,70	6,00
S8	31	M	185	67	60	2/2	7,79	4,75
S9	30	M	180	84	15	2/2	8,22	4,17
S10	22	M	183	76	60	2/2	10,71	6,50
S11	35	F	160	61	30	2/2	6,67	3,67
S12	25	M	175	72	60	2/2	6,65	11,40
S13	22	F	169	52	30	3/2	9,55	3,53
S14	24	F	150	53	30	3/3	11,36	1,40
S15	24	F	170	77	30	3/3	9,26	5,70
S16	23	F	170	57	60	2/2	8,85	5,09
S17	24	F	173	76	60	2/2	10,71	7,33
S18	26	M	178	79	15	2/2	9,38	7,33
S19	33	M	187	89	30	2/2	7,79	4,00
S20	23	F	171	57	60	2/2	7,41	4,50
S21	24	M	182	72	60	2/3	10,53	1,47
S22	22	F	162	52	15	2/2	7,14	7,83
S23	23	M	166	54	15	2/2	8,15	4,16
S24	24	F	167	85	30	2/2	7,21	1,00
S25	29	M	182	72	30	2/2	12,77	5,50
S26	24	F	163	65	15	2/2	9,29	5,06
S27	25	F	168	55	15	2/2	8,20	6,04
S28	23	F	168	51	15	2/2	9,52	5,00
S29	25	F	160	57	30	3/3	10,71	6,50
S30	25	F	158	53	60	2/2	11,11	1,83
S31	29	M	166	64	30	2/2	9,52	6,17
S32	30	M	186	72	60	2/2	7,23	4,67
S33	26	F	158	55	60	2/2	10,53	1,83
S34	29	F	168	61	30	2/2	6,25	6,33
S35	25	M	180	64	30	2/2	9,23	6,29
S36	24	M	176	74	30	2/2	11,32	5,33
Mean	26		171	66	37		9,15	5,07
Standard deviation	4		9	11	17		1,61	2,19

For each subject: stereoacuity (TNO) and visual acuity at close distance (Parinaud, left eye / right eye). Normal values are <100 for TNO, <3 for Parinaud and 9.5 ± 2 for the amplitude of accommodation.

### Postural and oculomotor data collection

In order to test our hypotheses (postural–hyp.1 –and oculomotor–hyp.2, hyp.3), the subjects stood on a force platform in front of a diode-table and wore an eyetracker.

The force platform (produced by TechnoConcept, Céreste, France and using the Standards of the Association Française de Posturologie) used to measure postural performance consists of two dynamometric clogs placed in a standardized position: feet placed side by side, forming a 30° angle with heels separated 4 cm. Each clog holds 2 force sensors (one beneath the metatarsal heads, one beneath the heel) which are force—electric tension transducers. The height and weight of the subjects were factored into the calculations of the CoP displacements. The CoP displacements were recorded over a period of 51.2s; the equipment contained an Analogue–Digital converter of 16 bits and the sampling frequency of the CoP was 40Hz.

**Fig 2:**
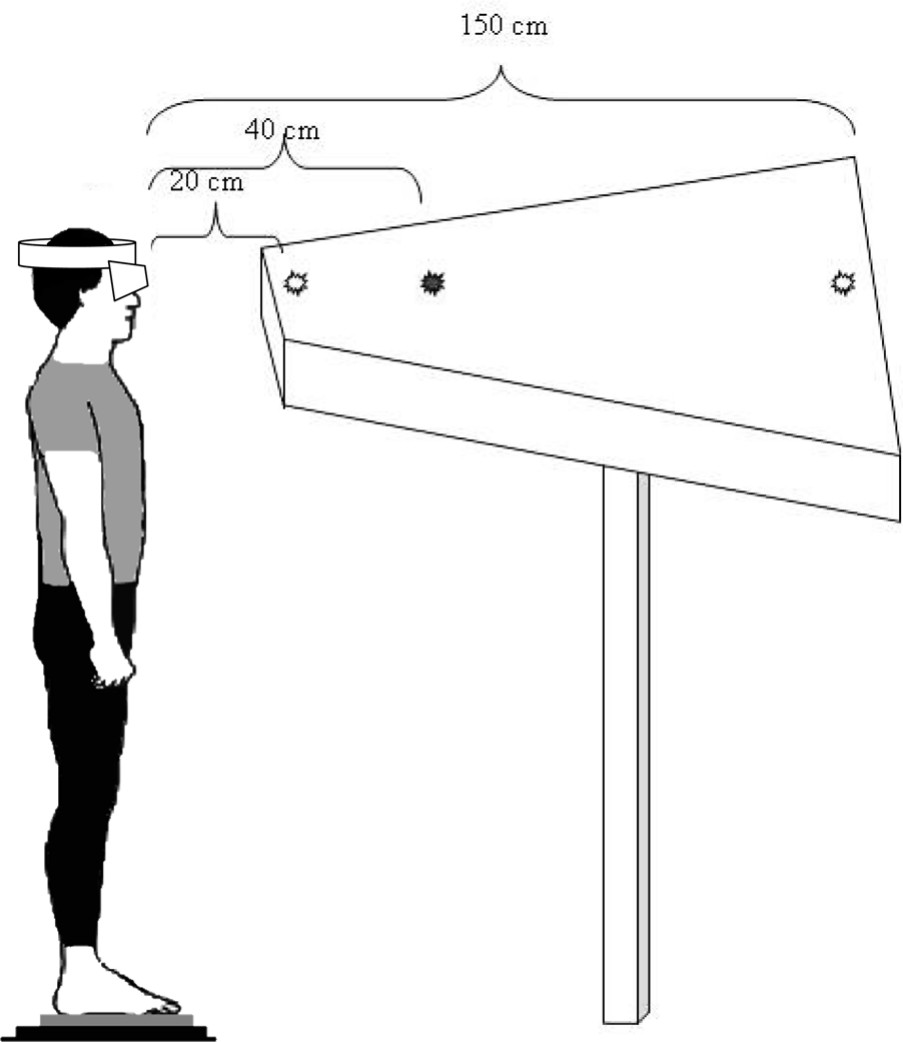
Visual target and eye movement recording. The subject is standing on a force platform, staring at the LEDs successively lit, which results in convergence and divergence eye movements.

The eye-tracker used to record eye movements was the Chronos Skalar video oculography apparatus. It consists of infra-red cameras, able to record eye movements in the dark, at a sampling of 200 frames per second [38]. (Fig 2). Such temporal resolution allowed us to record fine spatial and temporal parameters of dynamic vergence and saccades that we needed to test our oculomotor hypotheses (hyp.2, hyp.3).

### Testing conditions, postural and eye movement recording

The subjects were asked to stand still, barefoot, on the force plate in front of the diode-table set at eye level, the base of their nose aligned with the LEDs. The first LED is set at 20cm away from the subject's eyes, the second one at 40cm, and the third one at 150cm (Fig 2).

The measures were recorded in an experimental room normally furnished and dimly lit. The experiment was carried out in mesopic vision conditions so as to enable a good visibility of LED targets, favour focused attention and increase the recruitment of cutaneous afferents [39]. A sheet of paper was set beneath the subject’s feet in each condition. Three counterbalanced conditions of plantar stimulation were performed: no stimulation (Control condition), bilateral Medial Arch Support (MAS condition) and bilateral Lateral Arch Support (LAS condition) (Fig 1). We used 3 mm thick, shore rating of 60A and 250 kg/mm^3^ density, plantar inserts just like Janin and Dupui [7]. The mid-foot localization was chosen to be stimulated because it is known as a high-density zone of cutaneous feet mechanoreceptors with a high discriminating capability [40].

After a first familiarization trial, the eye movements and postural performances of each subject were recorded twice for each condition. To place the plantar inserts under the subjects’ feet in the right place, Betadine® was brushed under the mid foot of the subject after he lifted his heel. Then, he laid his heel on the sheet of paper, thus marking it with the Betadine® and lifted it again while the experimenter placed the insert on the medial half of the mark (MAS condition), or on the lateral half of it (LAS condition). This procedure was also done for the control condition, even if no insert was placed under the feet. In order to avoid a phenomenon of habituation of the sole cutaneaous mechanoreceptors, a two minute period of seated rest separated each recording [5].

After the calibration period (for each recording), during which the subjects performed horizontal saccades, the subjects were asked to alternate fixation between three LED targets. Those were placed at 20cm (angle of vergence = 18°), 40cm (angle of vergence: 9.23°), and 150cm (angle of vergence = 2.48°). For each trial the subjects started to fixate on the central LED (40cm) for 2400ms; then they had to fixate on the target LED which lit up either at 20cm, requiring for a convergence of 8.77°, or at 150cm, requiring a divergence of 6.75°. The target LED remained lit for 1700ms; the initial fixation LED switched off after an overlap period of 200ms. The subjects performed ten convergence and ten divergence movements, in a random order, coming back to the central LED at 40cm after each movement; trials were separated by blank periods of 500ms. This procedure was done twice for each plantar stimulation condition. The mean values of these eye movement recordings were used.

### Postural parameters and frequency analysis

To assess the effects of thin plantar inserts upon postural control, we analyzed the classical postural parameters: the Surface of CoP excursions, the antero-posterior (Y) position, the standard deviations of lateral (SDx) and antero-posterior (SDy) of body sways and the variance of speed of CoP (at the sampling frequency of 40Hz). The Surface area of the CoP represents 90% of the instantaneous positions of the CoP included within the confidence ellipse [41], eliminating the extreme points.

In order to better distinguish the effects of the two inserts (MAS and LAS), we also resorted to the new methods of analysis of the CoP displacements: the wavelet non linear analysis on fore-aft (y) and mediolateral (x) data, and the fractal analysis. Just like in the traditional approach, three frequency bands are used to classify postural sway: 0.05–0.5 Hz (F1), 0.5–1.5 Hz (F2), higher than 1.5 Hz (F3). Those frequency bands are considered to correspond to the use of different means of postural control: 0–0.5 Hz: visual-vestibular [42, 43], 0.5–1.5 Hz: cerebellar [43], beyond 1.5 Hz: reflexive loops [44, 45]. The main advantage of this analysis is to provide information on time in addition to the power and frequency provided by the classical FFT approach [46]. This proved more sensitive than the latter [44], especially in regards to the interpretation of the frequency peaks, which are not constant over time [46].

The Power Indices represent the amount of energy spent during the task, while the Canceling Time represents the total time during which the spectral power of the body sway for the frequency range is cancelled by the posture control mechanisms. Thus, a longer Canceling Time denotes a more efficient postural control [44, 46]. The postural instability index (PII) quantifying the postural performance, taking into account the two precedent indices (PI and CT), was also calculated as the following: PII = ∑_x_ ∑_y_ PI (F1, F2, F3) / CT (F1, F2, F3) [44, 46]. In healthy adults and during the single quiet stance task the PII is close to unity. This additional analysis and associated parameters were obtained with the software PosturoPro (Framiral, Cannes, France, www.framiral.fr).

The fractal analysis (FRAC) provides another complementary clue that allows us to get a more complete description of the postural performance of a standing subject. The fractal dimension (Hausdorff’s points) enables us to know whether two consecutive sampling points the stabilometric signal are correlated, i.e., linked by a causal relationship, or not, i.e. random, or stochastic movement. The PosturoPro software calculates the proportion of these uncorrelated points. A high percentage of these points is related to a physiological noise that does not need any postural adjustment. Conversely, a low percentage of Hausdorff’s reveals a causal relationship between the events that traduces an active control of the postural system [46, 47]. Moreover, according to several authors [48, 49], the fractal dimension of CoP is a very sensitive and quite reliable measure for assessing quiet stance balance, as well as a more accurate way than traditional parameters to detect postural adaptations during modifications of sensory inputs or environment [46]. As the stimulation of the medial part of the foot sole favors inversion [6, 28], which requires an active participation of the invertor muscles to oppose the natural trend to pronation [50], we assume that the percentage of uncorrelated points will be lower with the MAS with respect to Control and to LAS. That parameter is therefore useful to distinguish the effects of the inserts and propose an explanation of their physiological action (hyp.1).

### Eye movement data analysis

**Fig 3:**
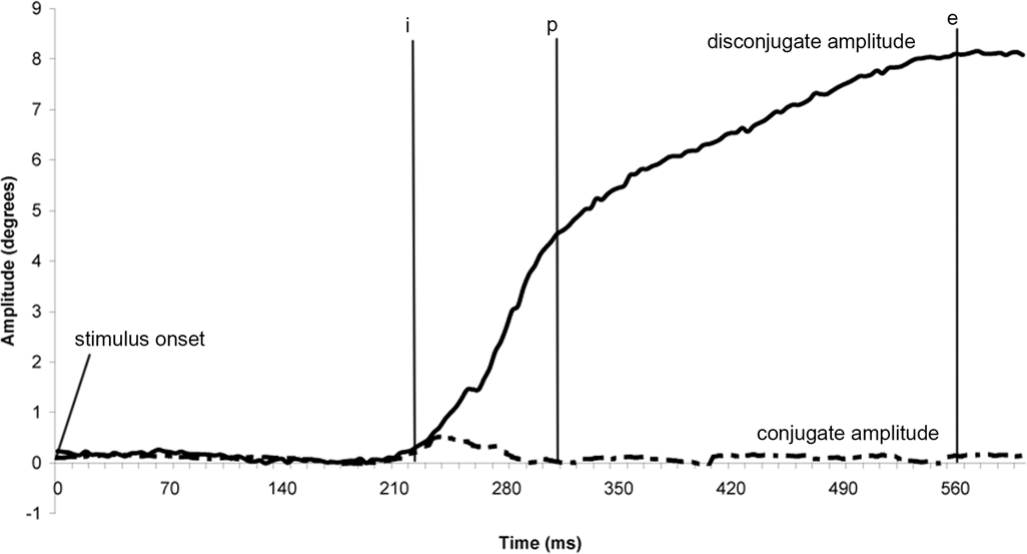
Typical recording of a convergence eye movement. Markers ‘i’ and ‘e’ indicate the onset and the offset of vergence eye movement based on the criterion of 5°/s velocity threshold. Marker ‘p’ indicate the separation between the phasic and tonic part of the movement. Full line: disconjugate amplitude; dotted line: conjugate amplitude.

**Fig 4:**
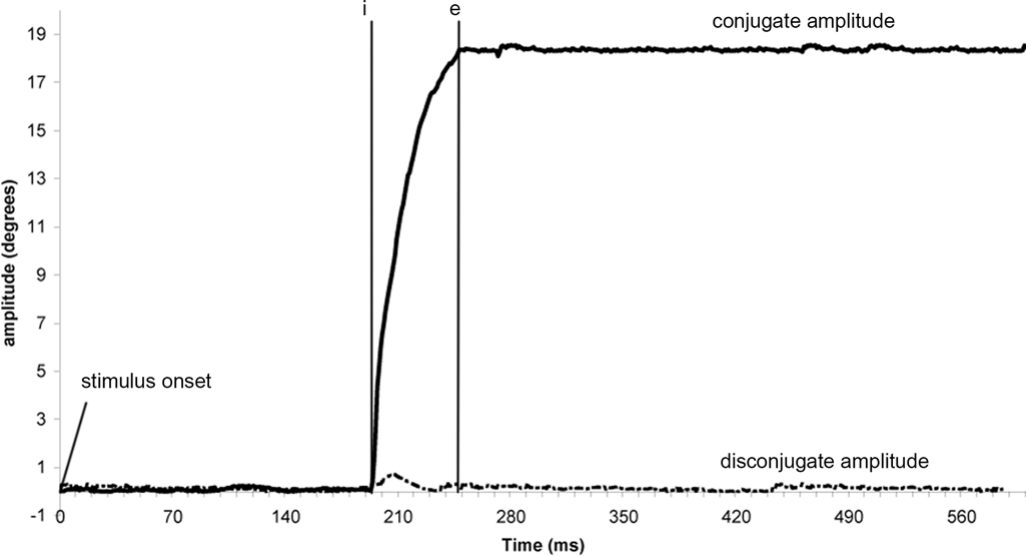
Typical recording of a saccade eye movement. Markers ‘i’ and ‘e’ indicate the onset and the offset of vergence eye movement based on the criterion of 45°/s velocity threshold. Full line: conjugate amplitude; dotted line: disconjugate amplitude.

The Chronos system gives two individual calibrated eye position signals, from which we calculated the conjugate signal, e.g. the mean of both eyes signals [(left eye + right eye)/2] and the disconjugate signal, e.g. the difference between the two eyes (left eye–right eye). After conducting low-pass filtering with a Gaussian finite impulse response filter (gain 0.1 at 85Hz), the eye velocity of either conjugate (saccades) or disconjugate (vergence) signal was computed using a symmetrical two-point differentiator. Horizontal saccades from the calibration period were also analysed. The detection of either saccades or vergence was performed using these calibrated conjugate, disconjugate or velocity signals. The beginning and the end of the eye movements were defined in terms of the precise moment in time when the eye velocity exceeded or dropped below 5°/s for vergence (see ‘i’ and ‘e’ markers, Fig 3), or 45°/s for saccades (see ‘i’ and ‘e’ markers, Fig 4). These criteria are standard [51, 52].

The total amplitude of vergence was measured as the difference between ‘i’ and ‘e’ positions (in degrees). The difference between ‘i’ and ‘p’ indicated the phasic component of the movement (i.e. its first 80 milliseconds—e.g. [26]). That part is the period for which no visual feedback can intervene in the execution of the movement because of afferent-efferent delays. Thus the phasic component is done under open-loop control. The tonic component was measured as the difference between ‘p’ and ‘e’ (see Fig 3) and corresponds to the closed-loop part of the movement, under the influence of visual retroaction. This phasic-tonic subdivision applies even for vergence along the median plane [26, 53–57].

The markers i’, ‘p’ and ‘e’ were also projected on the conjugate (Left + Right eye traces / 2) eye position signal that indicated the conjugate component occurring during vergence. For saccades (see Fig 4) the amplitude was measured as the difference (in degrees) between ‘i’ and ‘e’ positions. We also measured the disconjugate movement during the saccade by projecting the ‘i’ and ‘e’ markers on the disconjugate signal (Left—Right eye traces).

For all the eye movements (convergence, divergence, saccades) we also measured the latency (i.e. the time between stimulus onset and initiation of the eye movement), the duration (time between ‘i’ and ‘e’ markers), the maximum and the mean velocity (ratio amplitude/duration). We rejected eye movements in the wrong direction, contaminated by blinks, with latency shorter than 80ms (anticipation) or longer than 500ms for vergence, or 1000ms for saccades.

### Statistical analysis

Statistical analysis was performed using Friedman’s ANOVA (procedure of Statsoft / Statistica, release 7.1) since the test of Shapiro-Wilk revealed that some of the distributions were not normal and proved impossible to normalize.

Post hoc comparisons were done whenever necessary using the test of Wilcoxon, with P<0.05 considered as significant. The magnitude of the difference were assessed by the effect size (Cohen’s d) where the difference was considered either small (0.2 < d < 0.5), moderate (0.5 < d < 0.8), or large (d > 0.8) [58].

## Results

The results which concern the first hypothesis (postural–hyp.1) are shown in Table 2, which summarizes the means, standard errors and p values of the different postural parameters for each testing condition (C, MAS, LAS). Those parameters are the Surface of the CoP excursions, the X and Y position, SDx, SDy, the variance of speed, PII, PI, CT and FRAC for each plane (x and y) and for each frequency band (0.05–0.50 Hz, 0.50–1.50 Hz and 1.50–10.00 Hz).

**Table 2:** Postural results and p value of Friedman's ANOVA.

Postural parameters	Control	MAS	LAS	p value
**Surface of CoP (mm2)**	113,85 ± 37,44	89,88 ± 25,76	98,30 ± 26,53	**0,000**
SDx (SD of lateral sway, mm)	2,47 ± 0,71	2,34 ± 0,59	2,36 ± 0,57	0,518
SDy (SD of antero-posterior sway, mm)	3,50 ± 0,90	3,19 ± 0,71	3,22 ± 0,89	0,057
**Variance of speed (mm2/s2)**	17,94 ± 6,57	15,65 ± 4,23	15,86 ± 5,66	**0,009**
**Y (mean Y position of CoP, mm)**	14,58 ± 6,49	11,38 ± 5,75	12,74 ± 6,27	**0,001**
X (mean X position of CoP, mm)	6,06 ± 1,25	5,16 ± 1,53	4,83 ± 1,49	0,641
Wavelets PII	0,94 ± 0,29	0,90 ± 0,27	0,92 ± 0,30	0,558
Wavelets PIx (mm2*106) 0–0.5 Hz	60,51 ± 6,50	59,71 ± 5,43	60,17 ± 5,22	0,278
Wavelets PIx (mm2*106) 0.5–1.5 Hz	48,41 ± 5,94	47,94 ± 5,28	47,61 ± 5,09	0,156
Wavelets PIx (mm2*106) >1.5 Hz	30,71 ± 6,25	30,11 ± 6,00	29,69 ± 5,27	0,169
Wavelets PIy (mm2*106) 0–0.5 Hz	66,16 ± 4,91	65,92 ± 4,13	65,74 ± 5,25	0,558
Wavelets PIy (mm2*106) 0.5–1.5 Hz	54,78 ± 4,56	54,62 ± 3,61	54,54 ± 4,28	0,823
Wavelets PIy (mm2*106) >1.5 Hz	36,57 ± 3,73	35,99 ± 3,68	36,15 ± 4,69	0,895
**Wavelets CTx (s) 0–0.5 Hz**	1,00 ± 0,37	1,19 ± 0,38	1,04 ± 0,31	**0,021**
Wavelets CTx (s) 0.5–1.5 Hz	0,41 ± 0,26	0,31 ± 0,13	0,40 ± 0,24	0,184
Wavelets CTx (ms) >1.5 Hz	0,11 ± 0,05	0,11 ± 0,03	0,11 ± 0,02	0,558
Wavelets CTy (s) 0–0.5 Hz	0,78 ± 0,28	0,85 ± 0,34	0,83 ± 0,33	0,590
Wavelets CTy (ms) 0.5–1.5 Hz	0,70 ± 0,31	0,70 ± 0,28	0,66 ± 0,30	0,823
**Wavelets CTy (ms) >1.5 Hz**	0,12 ± 0,05	0,11 ± 0,05	0,10 ± 0,02	**0,032**
FRACx (% of uncorrelated points)	4,76 ± 2,35	4,59 ± 2,43	5,07 ± 2,59	0,264
**FRACy** (% of uncorrelated points)	2,27 ± 1,15	1,95 ± 1,17	2,18 ± 1,03	**0,013**

Means and standard errors of postural parameters for each condition.

p values of Friedman's ANOVA on the postural parameters, the significant ones are in bold letters. The significant post hoc tests are recorded in the text.

The results concerning the oculomotor hypotheses (hyp.2, hyp.3) are shown in Table 3, which summarizes the means, standard errors and p values of the non significant eye movements parameters for each condition. The significant ones are recorded in the text and in Tables D and E, in S2 Dataset. Eye movement results.

**Table 3:** Eye movement results and p value of Friedman's ANOVA.

Eye movement parameters	Control	MAS	LAS	p value
DIVERGENCE				
Conjugate amplitude	0,28 ± 0,02	0,32 ± 0,03	0,32 ± 0,03	**0,000**
Maximum velocity	67 ± 4	66 ± 4	67 ± 4	**0,000**
Mean velocity	10 ± 0	10 ± 0	10 ± 0	**0,000**
Latency	208 ± 6	211 ± 6	211 ± 6	**0,000**
Duration	431 ± 18	429 ± 18	433 ± 18	0,343
CONVERGENCE		** **		
Total amplitude (disconjugate)	5,43 ± 0,26	5,52 ± 0,25	5,58 ± 0,23	**0,000**
Phasic amplitude (disconjugate)	1,67 ± 0,10	1,69 ± 0,10	1,61 ± 0,08	**0,000**
Conjugate amplitude	0,47 ± 0,04	0,48 ± 0,05	0,43 ± 0,04	**0,000**
Maximum velocity	49 ± 3	51 ± 3	50 ± 3	**0,000**
Mean velocity	13 ± 1	14 ± 1	13 ± 1	**0,000**
Latency	186 ± 4	186 ± 4	190 ± 5	**0,000**
Duration	452 ± 14	447 ± 16	457 ± 17	0,343
SACCADES				
Conjugate amplitude	17,62 ± 0,30	17,47 ± 0,21	17,60 ± 0,14	0,256
Disonjugate amplitude	0,77 ± 0,06	0,71 ± 0,06	0,77 ± 0,06	0,169
Maximum velocity	429,13 ± 10	427,22 ± 8	426,57 ± 8	0,184
Mean velocity	264,32 ± 5	264,95 ± 4	263,07 ± 4	0,590
Latency	179,01 ± 5	175,12 ± 4	178,88 ± 5	0,895
Duration	66,91 ± 1	67,00 ± 1	67,42 ± 1	0,529

Means and standard errors of the non significant eye movements parameters for each condition, the significant ones are recorded in the text.

p values of initial and post hoc tests on the eye movements parameters for each condition, the significant ones are in bold letters. The significant post hoc tests are recorded in the text.

### Classical postural parameters results

The Friedman’s ANOVA performed on the Surface of the CoP excursions values showed a main effect of the sole stimulation conditions (Chi2 ANOVA_(36,2)_ = 17.39, *P <* .001).

**Fig 5:**
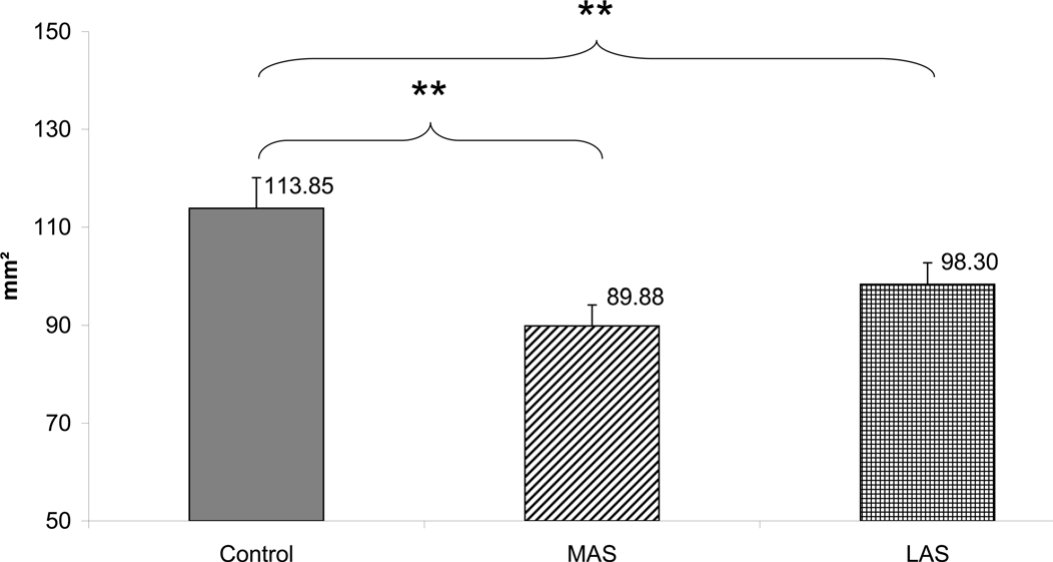
Area of CoP (mm²). Mean of the Surface area of the Center of Pressure excursions for each testing condition. Error bars represent the standard error. Asterisks indicate significant differences, with *P<0.05; **P<0.01.

The test of Wilcoxon showed a statistically significant difference of the Surface with MAS (z = 3.27, d = 0.75, *P* = .001) and LAS (z = 3.02, d = 0.48, *P* = .003) plantar stimulation as compared to the Control condition. Both sole stimulations inducing a decrease of the sway area (see Fig 5).

**Fig 6:**
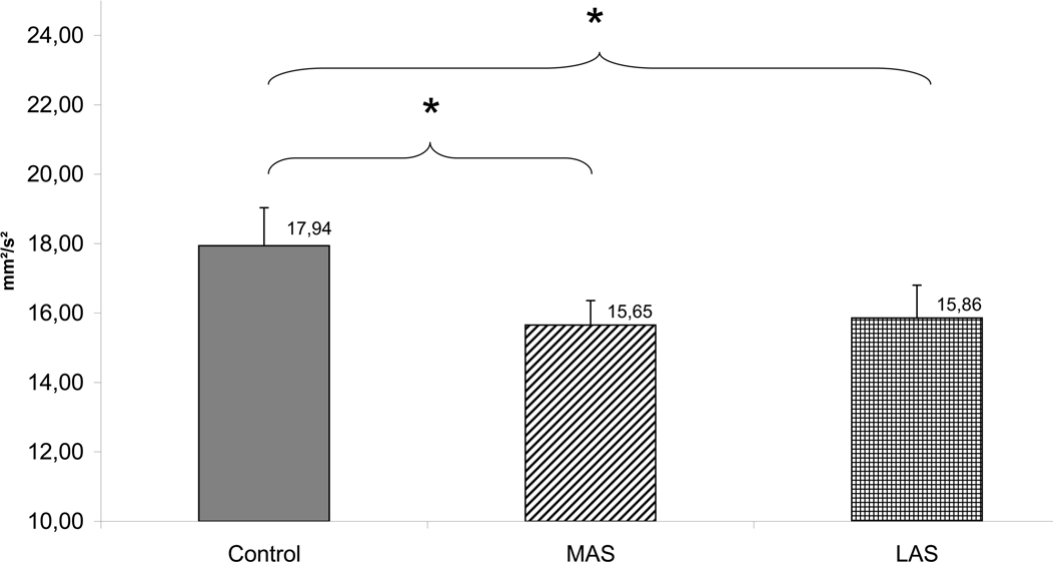
Variance of speed (mm²/s²). Mean of the Variance of speed for each testing condition. Error bars represent the standard error. Asterisks indicate significant differences, with *P<0.05.

In addition to this spatial effect, a Friedman’s ANOVA also revealed a main effect of the sole stimulations on the Variance of speed (Chi2 ANOVA_(36,2)_ = 9.39, *P =* .009). The test of Wilcoxon showed a statistically significant difference of that Variance of speed with MAS (z = 2.37, d = 0.41, *P* = .018) and LAS (z = 2.47, d = 0.34, *P* = .014) as compared to the Control condition. Both stimulations inducing a decrease of the Variance of speed (see Fig 6).

**Fig 7:**
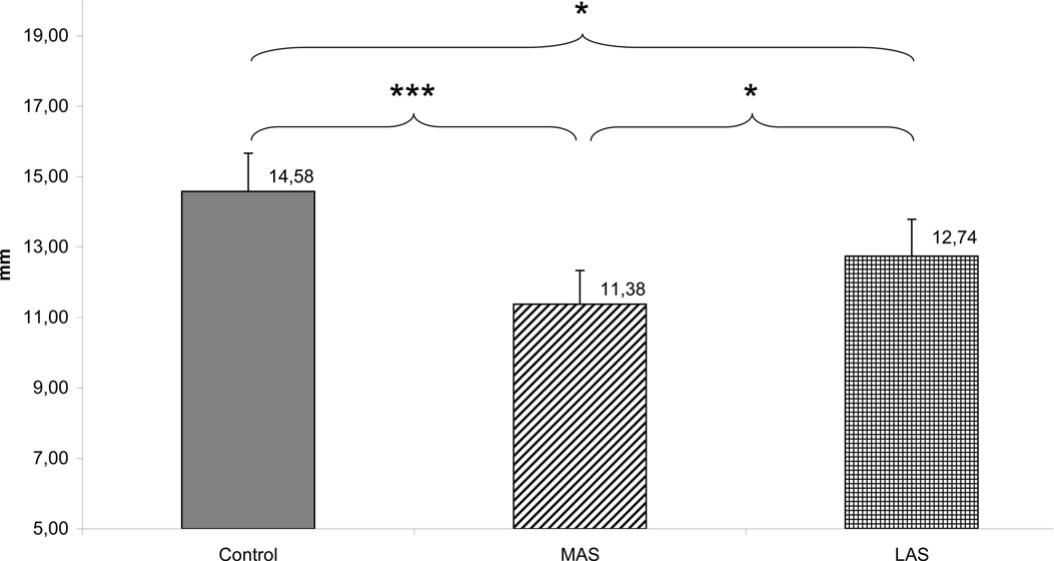
Y (mm). Mean of the Y position of the CoP for each testing condition. Error bars represent the standard error. Asterisks indicate significant differences, with *P<0.05; **P<0.01, and ***P<0.001.

Furthermore, interesting results appeared in regards to the position of the CoP: a Friedman’s ANOVA a main effect of the sole stimulation conditions on the antero-posterior (Y) position of CoP (Chi2 ANOVA_(36,2)_ = 15.17, *P =* .001). The test of Wilcoxon showed a statistically significant difference between Control and MAS (z = 3.82, d = 0.52, *P* < .001), with MAS more posterior than Control; between Control and LAS (z = 2.56, d = 0.29, *P* = .010), with LAS more posterior than Control; and between MAS and LAS (z = 2.12, d = 0.23, *P* = .034), with MAS more posterior than LAS (see Fig 7).

### Wavelet and fractal results

In regards to the parameters elaborated from the wavelet transform, a Friedman’s ANOVA showed a main effect of the sole stimulations on the Cancelling Time of the first frequency band (Chi2 ANOVA_(36,2)_ = 7.72, *P* = .021) on the mediolateral sway, and a Friedman’s ANOVA showed a main effect of the sole stimulations on the Cancelling Time of the third frequency band (CTy3), (Chi2 ANOVA_(36,2)_ = 6.86, *P* = .032) on the anteroposterior sway.

**Fig 8:**
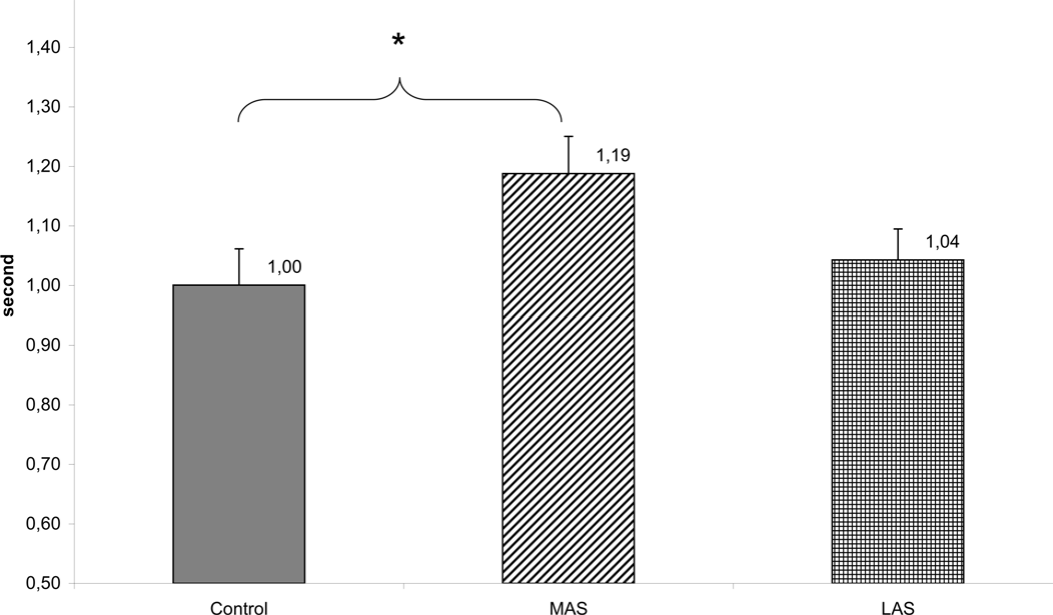
Wavelets CTx1 (s) 0–0.5 Hz. Mean of the Cancelling Time on the mediolateral sway of the first frequency band for each testing condition. Error bars represent the standard error. Asterisks indicate significant differences, with *P<0.05.

The test of Wilcoxon showed a statistically significant difference of the cancelling time of the first frequency band on the mediolateral sway between MAS and Control (z = 2.37, d = 0.51, *P* = .018). The CT1x was longer with the MAS with respect to Control. (see Fig 8)

**Fig 9:**
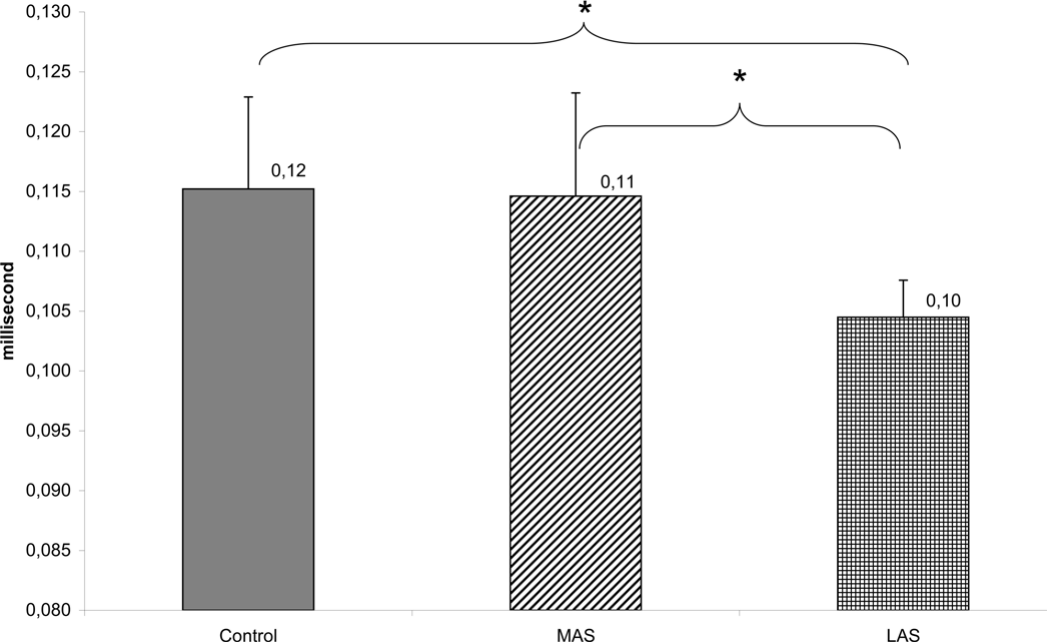
Wavelets CTy3 (ms) >1.5 Hz. Mean of the Cancelling Time on the anteroposterior sway of the third frequency band for each testing condition. Error bars represent the standard error. Asterisks indicate significant differences, with *P<0.05.

The test of Wilcoxon showed a statistically significant difference of the cancelling time of the third frequency band on the anteroposterior sway between Control and LAS (z = 2.51, d = 0.34, *P* = .012), and between MAS and LAS (z = 2.14, d = 0.28, *P* = .032). The CTy3 was longer in the Control and MAS condition as compared to LAS (see Fig 9).

Finally, a Friedman’s ANOVA showed a main effect of sole stimulations on the fractal analysis on the anteroposterior sway (Chi2 ANOVA_(36,2)_ = 8.67, *P* = .013).

**Fig 10:**
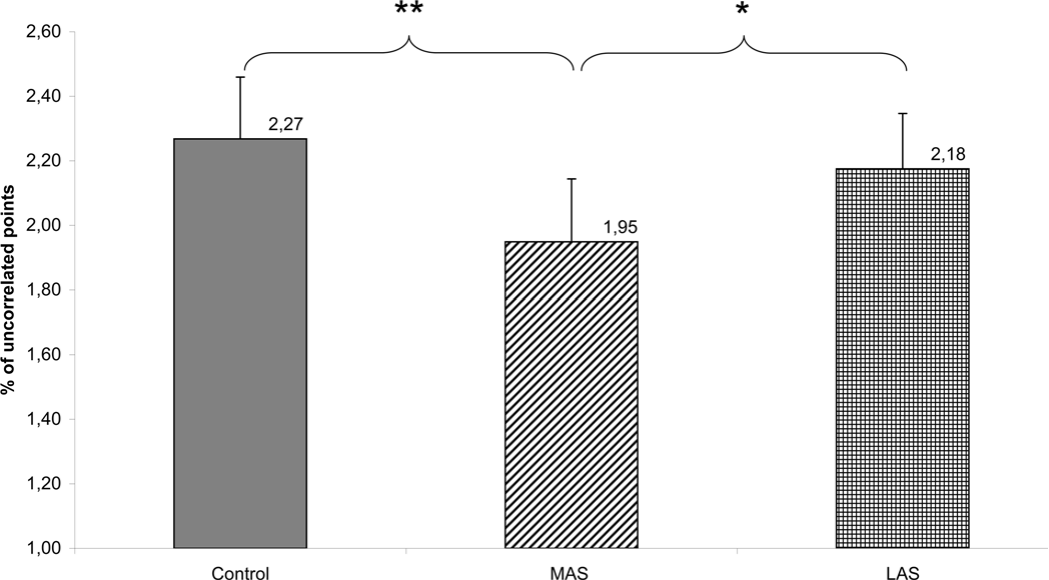
FRACy. Fractal analysis of the Center of Pressure displacements on the anteroposterior axis for each testing condition. Error bars represent the standard error. Asterisks indicate significant differences, with *P<0.05; **P<0.01.

The test of Wilcoxon showed a statistically significant difference of the percentage of uncorrelated points on the anteroposterior sway between MAS and Control (z = 2.89, d = 0.27, *P* = .004), and between MAS and LAS (z = 2.27, d = 0.21, *P* = .023). The percentage being lower with the MAS with respect to Control and to LAS. (see Fig 10)

### Vergence results

#### Divergence

**Fig 11:**
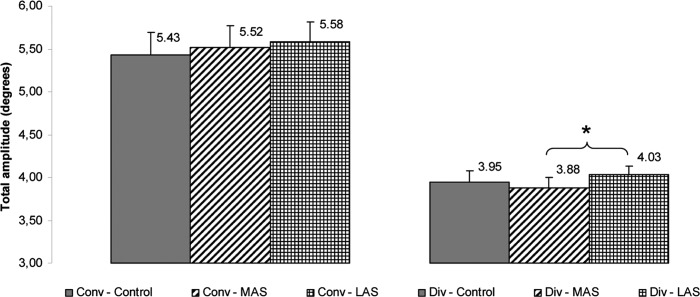
Total amplitude. Mean of the total amplitude of convergence and divergence for each testing condition. Error bars represent the standard error. Asterisks indicate significant differences, with *P<0.05.

On the total amplitude, the Friedman’s ANOVA showed a main effect of the sole stimulation conditions (Chi2 ANOVA_(36,5)_ = 105.60, *P <* 0.001). The test of Wilcoxon showed a statistically significant difference of the total amplitude between MAS and LAS (z = 2.07, d = 0.21, *P =* 0.038), the amplitude being higher with LAS. (see Fig 11).

**Fig 12:**
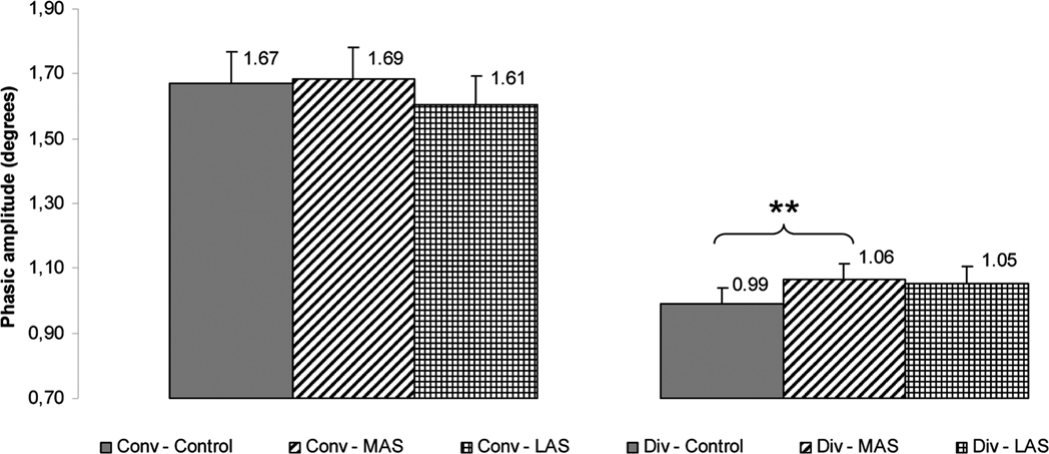
Phasic amplitude. Mean of the phasic amplitude of convergence and divergence for each testing condition. Error bars represent the standard error. Asterisks indicate significant differences, with *P<0.05; **P<0.01.

Then, a Friedman’s ANOVA was performed on the phasic amplitude of divergence and showed a main effect of the sole stimulation conditions (Chi2 ANOVA_(36,5)_ = 90.75, *P <* 0.001). The test of Wilcoxon showed a statistically significant difference of the phasic amplitude between MAS and Control (z = 2.84, d = 0.25, *P =* 0.004), the amplitude being higher with MAS. (see Fig 12).

**Fig 13:**
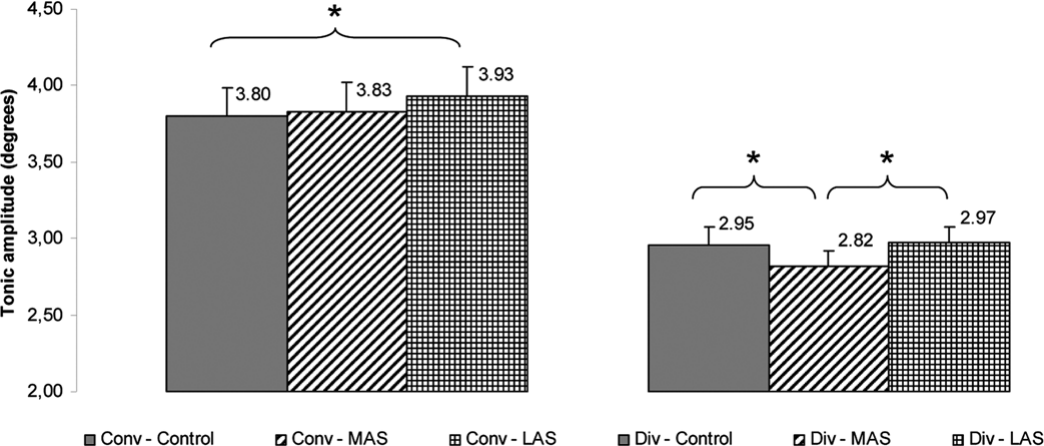
Tonic amplitude. Mean of the tonic amplitude of convergence and divergence for each testing condition. Error bars represent the standard error. Asterisks indicate significant differences, with *P<0.05.

Another Friedman’s ANOVA was done on the tonic amplitude of divergence and showed a main effect of the sole stimulation conditions (Chi2 ANOVA_(36,5)_ = 72.92, *P <* 0.001). The test of Wilcoxon showed a statistically significant difference of the tonic amplitude between MAS and Control (z = 2.26, d = 0.21, *P =* 0.024) and between MAS and LAS (z = 1.96, d = 0.25, *P =* 0.050), the amplitude being lower with MAS as compared to the two other conditions (see Fig 13).

#### Convergence

On the tonic amplitude of divergence a Friedman’s ANOVA showed a main effect of the sole stimulation conditions (Chi2 ANOVA_(36,5)_ = 72.92, *P <* 0.001). The test of Wilcoxon showed a statistically significant difference of the tonic amplitude between LAS and Control (z = 2.09, d = 0.12, *P =* 0.037), the amplitude being higher with LAS. (see **Fig 13**).

#### Convergence / divergence with the same plantar inserts comparisons

For clarity reasons those secondary results are available in S2 Dataset. Eye movement results.

### Saccades results

There were no significant differences between the tested conditions on any of the saccade parameters.

## Discussion

The major finding concerning postural control is an improvement of body balance through spatial and temporal parameters with both stimulations, which confirms our first hypothesis. An additional observation is that the Y position of the CoP gets more posterior relative to control, particularly with the MAS.

As regards oculomotor control, the most important result is that thin plantar stimulations do have an effect only upon vergence, confirming our second hypothesis. We believe it is the first time that such an effect is observed by objective measures.

### Effects on postural control

#### Decrease of Surface and Variance of Speed

The plantar inserts improve postural stability (decrease of Surface), confirming hyp.1. The variance of speed also decreases, suggesting a lower energy cost [32]. These results can first be explained by the enhancement of the cutaneous afferents fed to the postural system because of the 3-millimetres difference of height created by the arch supports and detected by the numerous mechanoreceptors of the plantar skin [40].

The experiments of Kavounoudias [15, 17] showed that the foot sole behaves like a “dynamometric map” that quantifies the repartition of plantar pressures. This property allows the CNS to adjust the muscle tone, resulting in whole-body tilts of appropriate amplitude in the opposite direction which cancel the difference of plantar pressure and compensate the perceived disequilibrium. According to these authors, the repartition of plantar pressure is therefore a very precious clue for the CNS to assess the importance of the deviation of the body relative to its vertical reference, and thus enables it to reduce that gap.

An abstract of Janin and Toussaint [9] suggested that a small number of stimulations have beneficial effects upon postural control. Our results are in line with theirs. Furthermore, in the experiment of Janin the subjects were staring straight ahead, whereas in this one they were performing eye movements. While the eyes are moving, thin plantar stimulations still have an influence upon postural control despite the enrichment of the proprioceptive eye-muscle afferents [21, 23]. Hence, the increased use of this signal does not seem to lower the importance of the plantar afferents in quiet stance balance regulation [16, 17].

Yet, as regards the specific effects of medial and lateral stimulations, unlike the latter authors, we did not notice any difference in the decrease of Surface and Variance of Speed between them. It may be a matter of density of cutaneous mechanoreceptors: the lateral region of the fore-foot stimulated by Janin is richer in three of the four types of receptors (SAI, SAII, FAI) than the medial region, whereas the midfoot chosen in this study has the same density of receptors in both parts [40].

There are some reasons to think that plantar inserts can also act on posture by indirect ways. Indeed, plantar cutaneous afferents implement not only short reflexive loops [5, 7, 28] but also medium and long, supra-spinal loops [17, 59]. Moreover, several authors report information exchanges between the plantar and visual inputs of the postural system in the superior colliculus [60], or the cerebellum [61]. Here, the benefit of cutaneous afferents stimulation upon balance persisting during eye movement (see above) suggests a synergic cooperation of both inputs (plantar and visual) to ensure postural control, as it was already suggested by Roll and Roll [1, 2]. The hypothesis for some indirect action also comes from the effects observed on vergence amplitudes, which are changing with MAS and LAS as compared to Control—and contrary to saccades (see section ‘Effects of plantar stimulations upon vergence‘). Knowing that those movements have a positive influence upon posture [21, 23], it appeared plausible that the postural effects observed here would be secondary to those changes in vergence amplitudes. To answer this question, linear regression analyses were performed between the significantly changing parameters of posture and vergence. As no significant correlations were identified, it seems that the postural effects are only direct, via the sole mechanoreceptors.

Wavelets and fractal fine analysis give additional information about the effects of MAS and LAS. The Cancelling Time of medio-lateral sway in the low frequency band increases with MAS as compared with the no-stimulation condition, which denotes a more efficient postural control. On the contrary, the decrease of the Cancelling Time of antero-posterior body sway for the high range frequency band with the LAS tends to show a less efficient postural control with that stimulation as compared to the two other conditions. Finally, the fractal analysis suggests that MAS induces a more active control of posture compared to no-stimulation or LAS.

To explain these results (wavelets and fractal), we can hypothesise that the concept of dynamometric map held by Kavounoudias et al. [15, 17] can apply separately for each foot: with MAS, the medial part of the midfoot perceives an increase of pressure which entails the postural synergies enabling to cancel that difference of pressure, and results in a supination of the foot, and conversely, a pronation for the LAS. These results are in accordance with those of previous studies [6, 28]. The lateral part of the foot being considered as its stable part [62, 63], the supination of both feet would be responsible for better stability, with a more active participation of the invertor muscles needed to oppose the natural trend to pronation [50]. Convesely, the pronation provoked by the LAS would result in a more passive wedging on the medial, mobile part of the foot.

#### Position of CoP—clinical considerations

Our findings concerning the mean Y position of the CoP are consistent with the ones of Janin and Toussaint [9]: the plantar inserts, whether medial or lateral, entail a posteriorisation of the CoP and MAS has a more important action than LAS.

Both stimulations are indeed placed ahead of the talocrural axis and are therefore thought to simulate a forward whole-body tilt, compensated by a backward regulating response. The stronger result obtained with the MAS can also be explained by the inversion of the foot which it is correlated to whole-body posteriorisation [64]. These results are clinically relevant because the anteriorisation of posture is known to be injurious, in particular as concerns low-back pain and knee pain [29, 65, 66].

### Effects on oculomotor control

#### Effects of plantar stimulations upon vergence

**Fig 14:**
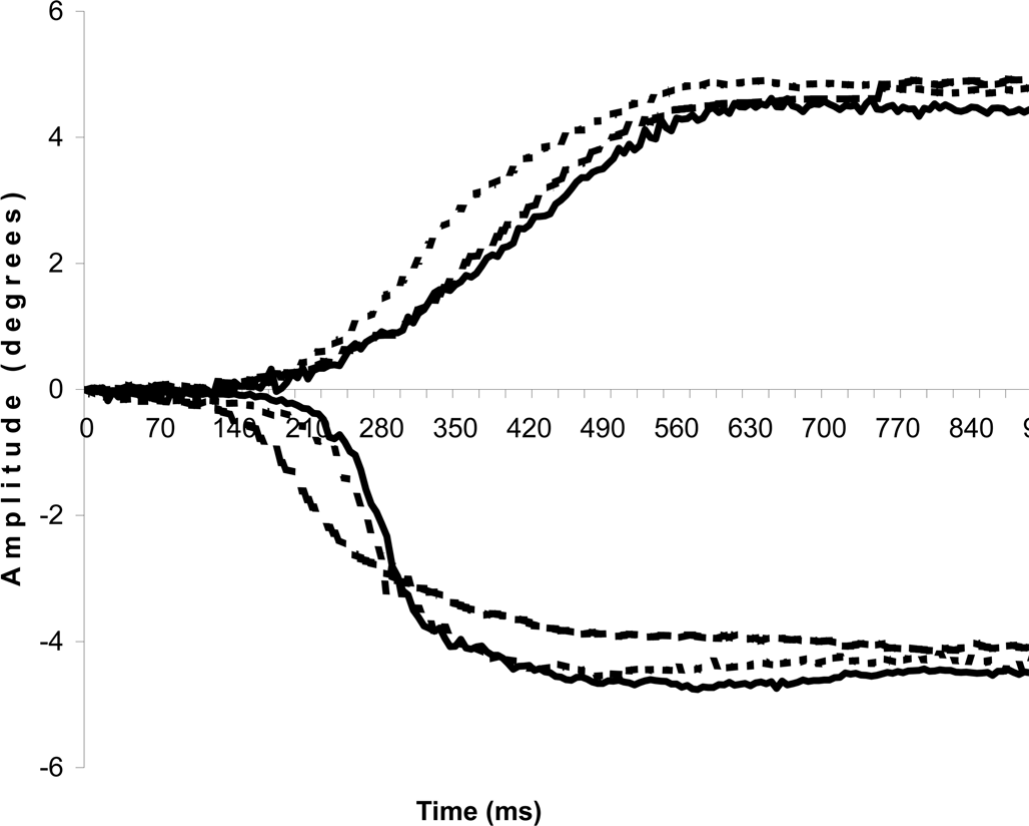
Example of convergence and divergence traces in the 3 testing conditions of one subject. Full line: control condition (no plantar insert), big-dotted line: with MAS, small-dotted line: with LAS.

Our second hypothesis is confirmed: mechanical stimulation of the foot sole by thin plantar inserts has an influence exclusively upon vergence, not on saccades. The other consideration concerning the synergic functioning of feet and eye muscles (hyp.3) is only partly confirmed: thin plantar inserts act in a different way depending on the part of the foot sole being stimulated, but not as simply as we thought. Indeed, we notice that there is no difference between the plantar stimulations and the control condition in regards to the total amplitude both of convergence or divergence. Yet, the examination of the two phases (phasic and tonic) of the vergence movements reveals the effects of the plantar stimulations. Divergence is more influenced than convergence and the two stimulations do not have the same action: MAS is more effective than LAS and acts upon divergence, whereas LAS produces its effects upon convergence only. See Fig 14 for an example of vergence movements in the 3 testing conditions.

MAS is responsible for a significant increase of the phasic amplitude of divergence as compared to Control, and conversely, a decrease of the tonic part of the eye movement. The total and tonic amplitude are also significantly lower with MAS than with LAS, confirming the different effects of those stimulations. LAS only causes an increase of the tonic amplitude of convergence. Theoretically these observations are compatible with the existing double-control model of vergence movement, with a two-phases organisation: a phasic, pre-programmed open-loop part, and then a tonic, closed-loop part under the influence of visual retroaction (see section ‘Eye movement data analysis‘). As MAS increases the pre-programmed proportion of the divergence movement and conversely decreases the tonic one, it seems to restrict the influence of visual retroaction in the control of divergence, thus resulting in a more programmed movement. Interestingly, LAS stimulation has an opposite effect upon convergence, resulting in the increase of its tonic amplitude. In other words, MAS renders divergence less visually driven, while LAS renders convergence more visually driven.

Those modifications of vergence movements following the manipulation of cutaneous plantar afferents can be explained by the existence of common command centres, or common zones of the CNS able to exchange information between plantar and visual inputs. Hollands et al. [61, 67–69] showed that there is a strong coupling in the execution of eye and feet movements of subjects rotating their trunk or walking. They propose that the cerebellum or the superior colliculus (both zones known to play a role in the control of vergence–e.g. [26]) could be such zones. To support this thesis, a fMRI study of Kavounoudias et al. [70] showed that the cerebellum is an area involved in multisensory integration, namely proprio-tactile.

The specificity of the effects of the plantar stimulations upon convergence and divergence movements suggests the existence of central mechanisms of actions that differ depending on the stimulated foot zone. They are also in line with studies putting forward the distinct control of convergence and divergence (e.g. [21]). For example, there are groups of neurons in the frontal eye field of the cortex that play a role in convergence which are different than the ones that take part in divergence [71]. Likewise, the anterior part of pre-frontal cortex is assigned to convergence, whereas the posterior part is assigned to divergence [72].

#### Convergence / divergence comparisons

For clarity reasons the discussion related to those secondary results is available in S2 Dataset. Eye movement results.

#### Effects of plantar stimulations upon saccades

Unlike vergence, saccades parameters of movement are not affected by the plantar stimulations, which confirms our second hypothesis. It is not surprising, knowing that saccade is a stereotyped, ballistic movement (500°/s, lasting around 50 milliseconds—Leigh and Zee 2006, see Fig 4) which does not seem compatible with the use of a distant source of information, implying, for the supra-spinal loops a latency around 100ms (Fallon et al. 2005). Another explanation is that some CNS zones like the supraoculomotor area of the mesencephalic reticular formation are specifically assigned to the control of vergence and might therefore be specifically influenced by the cutaneous afferents, contrary to the zones taking part only in the control of saccades (brainstem burst and omnipauses neurons, thalamus, basal ganglia—e.g. [26]).

Besides, despite common zones are also involved in conjugate and disconjugate eye movements, like the abducens nucleus, the lateral and medial recti of the oculomotor nucleus, the nucleus recticularis tegmenti pontis, the superior colliculus, the cerebellum, or some areas of the cerebral cortex (primary visual cortex, middle temporal cortex, medial superior temporal cortex, lateral intraparietal area, frontal eye field), the sensitivity to the modifications of eye position of the concerned neurons is not the same for vergence or conjugate movements (e.g. [26]).

These findings are also in congruence with the ones of other experiments which did not notice any modification of saccades parameters in seated compared to walking subjects (healthy or cerebellar) [73], nor after walk training among patients with cerebellar disorder [74].

## A Synthesis

To conclude, we show for the first time that feet tactile afferents influence both postural and oculomotor control and keep their predominant role in orthostatic balance regulation while the eyes are moving. Interestingly there is no tight correlation between posture and ocular vergence modulations by the plantar inserts even though both are influenced. This would mean that although under common influence and interaction the CNS uses the podal signal for postural control and the podal signal for vergence control via specific mechanisms. Our assumption that the lateral stimulation would entail a better postural control than the medial one is not supported by the results. Indeed, the Surface and Variance of Speed parameters of the CoP excursions do not show any difference between those stimulations, while a closer look upon the Y position, the wavelets and fractal analysis reveals that the MAS is more beneficial than the LAS. As regards eye movement, our initial hypothesis (hyp.2) is confirmed: the modification of plantar cutaneous afferents with thin plantar inserts has an action upon vergence only (not saccades). The modification of amplitude could be explained by exchanges of plantar and visual information in common zones of the CNS. The observed modifications are different depending on the stimulated foot zone.

A limitation of these findings could be the very low amplitude of the modifications observed (around 0.1°). Yet this effect, always small in amplitude, was systematic and statistically significant and is consistent with the results obtained by Popov et al. [12] after vibration of neck muscles (around 0.5°). Moreover, the movements of vergence are extremely frequent during daily life and small but repeated phenomena are thought to have a major impact on postural control [75–77].

As we recorded postural control in quiet stance, the head of the subjects was not fixated. Thus, changes in its position could lead to a change in vergence, like in the experiment of Erkelens et al. [13]. Yet, in that experiment, the subjects performed wide, voluntary fore-aft movements of the torso, while in ours they were instructed to stand still, in quiet stance. Therefore it does not seem likely that the observed changes in vergence could be attributed to changes in posture. Indeed, postural results did not show any statistical difference in the mean medio-latreal position of the CoP, the modifications of the antero-posterior sways of the CoP were only 2–3 millimetres, and it has been shown that forceplates measurements reflect the same properties of movements than actual body motions (namely for the hip, shoulder and head–[78]). Moreover, the specificity of the effect of each stimulation on vergence only also argues that the changes are mediated on the vergence system by the inserts and are not due to posture movement. We are planning to set up another experiment with a 3D motion analysis system that will answer that question.

As concerns the statistical analysis, we chose not to apply any p-value adjustments because the use of such methods is controversial [79, 80]. We followed Rothman’s point of view [81] who considers that these kinds of adjustments increase type II errors and risk to have the researchers miss possibly interesting findings. However, it is worth to note that if Bonferroni corrections for multiple comparisons had been used, the significant p-value threshold would have been of .017, so that some of the results would have failed to reach significance.

Finally, it is remarkable that the mean vergence amplitude values are rather hypometric. This could be due to being standing in conjunction with the absence of instruction from the investigator to force for high accuracy.

This experiment can have clinical implications: this way of increasing the plantar afferents is quite simple, it does not need any battery contrary to vibrating devices, and the thinness of foot orthoses resorting to such inserts makes them easier to wear than big-wedged insoles. Therefore it may be useful to prevent mechanical pain or pathologies correlated with postural deficiency [29–34, 82] provided those effects persist over time. An experiment of Perry et al. [83] showed that peripheric thin mechanical stimulation of the foot of older subjects improves their lateral walking stability on the long term (12 weeks). Further studies are required to evaluate if specific thin plantar inserts keep their effects in quiet stance on the long run too.

These findings can draw the attention of clinicians on the importance of foot sensibility in postural control and encourage them to be aware of the consequences of stimulating the plantar skin, even with inserts which could seem very thin. Clinicians using insoles or foot orthosis (podiatrists for example) should choose wisely the inserts they place beneath the feet of their patients. The therapists working on eye movements (ophtalmologists, orthoptists, optometrists) should also take in account the influence of the entire posture upon vergence capabilities. They should be well aware of the possible difference of influence of plantar afferents when they examine a seated or a standing patient.

This study is a first step that opens a new field for further experiments with synchronized eye movement and postural recordings and analysis (posture with fixation versus posture with convergence versus posture with divergence) on a moment to moment basis. They call to develop a multisensory integrative research approach which requires further technological developments.

## Supporting Information

S1 Dataset Postural results. (XLS)

S2 Dataset Eye movement results. (XLS)
